# Cognitive Function Is Impaired in Patients with Recently Diagnosed Type 2 Diabetes, but Not Type 1 Diabetes

**DOI:** 10.1155/2018/1470476

**Published:** 2018-08-09

**Authors:** Theresa van Gemert, Wolfgang Wölwer, Katharina S. Weber, Annika Hoyer, Klaus Strassburger, Nora T. Bohnau, Marie A. Brüggen, Katharina Ovelgönne, Eva-Maria Gössmann, Volker Burkart, Julia Szendroedi, Michael Roden, Karsten Müssig, GDS Group

**Affiliations:** ^1^Division of Endocrinology and Diabetology, Medical Faculty, Heinrich Heine University, Düsseldorf, Germany; ^2^Institute for Clinical Diabetology, German Diabetes Center, Leibniz Institute for Diabetes Research, Heinrich Heine University, Düsseldorf, Germany; ^3^German Center for Diabetes Research (DZD), Neuherberg, München, Germany; ^4^Department of Psychiatry and Psychotherapy, Medical Faculty, Heinrich Heine University, Düsseldorf, Germany; ^5^Institute for Biometrics and Epidemiology, German Diabetes Center, Leibniz Institute for Diabetes Research, Heinrich Heine University, Düsseldorf, Germany

## Abstract

**Objective:**

To test whether cognitive function is impaired in early states of diabetes and to identify possible risk factors for cognitive impairment.

**Methods:**

A cross-sectional analysis within the German Diabetes Study included patients with type 1 or type 2 diabetes within the first year after diagnosis or five years after study inclusion and metabolically healthy individuals. Participants underwent comprehensive metabolic phenotyping and testing of different domains of cognitive function. Linear regression models were used to compare cognition test outcomes and to test associations between cognitive function and possible influencing factors within the groups.

**Results:**

In participants with recently diagnosed diabetes, verbal memory was poorer in patients with type 2 diabetes (*P* = 0.029), but not in type 1 diabetes (*P* = 0.156), when compared to healthy individuals. Five years after diagnosis, type 2 diabetes patients also showed lower verbal memory than those with type 1 diabetes (*P* = 0.012). In addition to crystallized intelligence, a higher body mass index among individuals with recently diagnosed type 2 diabetes and male sex among individuals with recently diagnosed type 1 diabetes were associated with impaired verbal memory (all *P* < 0.05).

**Conclusion:**

Verbal memory is impaired in individuals with recently diagnosed type 2 diabetes and likely associated with higher body mass. This trial is registered with the trial registration number NCT01055093.

## 1. Introduction

Previous cross-sectional [1, 2] as well as longitudinal [3] studies showed that long-standing type 1 as well as type 2 diabetes mellitus is associated with a higher risk of impaired neurocognitive function and dementia. The underlying pathophysiological mechanisms are still largely unknown. Some clinical parameters, like elevated fasting blood glucose levels and hemoglobin A1c (HbA1c), as well as the presence of neuropathy or retinopathy, have been associated with impaired cognitive function [4–6]. However, not all studies indicated an association of elevated fasting blood glucose levels and cognitive dysfunction [7, 8]. Impaired cognitive function has been also associated with cardiovascular risk factors like hypertension, dyslipidemia, arteriosclerosis, or smoking in individuals with type 2 diabetes [1, 9, 10]. In contrast, other possible risk factors, like obesity, history of cardiovascular complications, or diabetes duration, have not shown any relationship with cognitive impairment in patients with type 1 or type 2 diabetes [8]. Also, the relationship between the occurrence of hypoglycemia and cognitive impairment is discussed controversially. Some, but not all studies, showed a correlation between frequency of hypoglycemia and cognitive impairment [11, 12]. Furthermore, hypoglycemia alone does not seem to be the primary factor determining cognitive impairment, but might increase the risk for cognitive decline in conjunction with microvascular complications [2].

Cognitive dysfunction among individuals with type 1 or type 2 diabetes appears to share many similarities, that is, mental and motor slowing, impaired attention, and executive function. However, unlike individuals with type 1 diabetes, those with type 2 diabetes often reveal deficits on measures of learning and memory [5]. In general, the majority of previous reports focused on a single type of long-standing diabetes and often assessed only single cognitive domains and functions.

The present study therefore aimed at comparing a broad range of cognitive functions between recently diagnosed individuals with type 1 or type 2 diabetes and metabolically healthy individuals using a comprehensive test battery. In addition, this study assessed parameters possibly influencing cognitive functions, that is, insulin sensitivity, inflammation, glycemic control, and overweight/obesity. We hypothesized that cognitive performance may be impaired in recently diagnosed type 2 diabetes, but not in recently diagnosed type 1 diabetes.

## 2. Materials and Methods

### 2.1. Study Population

Patients with diagnosed diabetes and healthy individuals participated in the prospective observational cohort study, German Diabetes Study (GDS; http://clinicaltrials.gov: NCT01055093) [13]. In brief, the GDS investigates the disease progression of recent-onset diabetes and the development of diabetes-associated complications to improve risk assessment and targeted treatment of patients with diabetes. Participants aged between 18 and 69 years with known diabetes duration of less than 12 months are eligible for study participation. Individuals undergo detailed metabolic phenotyping within the first year after clinical diabetes diagnosis as well as five years thereafter [13]. The main exclusion criteria for all individuals were acute infections, evidence of congestive heart failure, kidney diseases, liver diseases, psychiatric or addictive diseases, history of malignancies, or pregnancy. For healthy individuals, first-degree relatives with diabetes and/or impaired fasting glucose were the additional exclusion criteria. This analysis included all consecutive participants recruited at the German Diabetes Center from January 2013 to June 2016. The present study comprises a total of 353 individuals, of whom 201 individuals were recently diagnosed with type 1 or type 2 diabetes, 110 individuals had a mean known diabetes duration of five years, and 42 individuals were metabolically healthy. All individuals with type 1 diabetes were treated with insulin, whereas the majority of individuals with type 2 diabetes received oral glucose-lowering medications as therapy.

All individuals gave their written informed consent before participating in this study, which was performed according to the Declaration of Helsinki and approved by the ethics board of Heinrich Heine University Düsseldorf, Germany.

### 2.2. Cognition Tests

Cognition tests lasted around 45 minutes. Cognitive function was captured by a standardized test battery, that is, the Brief Assessment of Cognition in Schizophrenia (BACS) test [14], as well as tests investigating executive function, social cognition, and crystallized intelligence. All tests were conducted by a trained interviewer. BACS was chosen because it contains a comprehensive standardized test battery which includes tests of verbal memory, working memory, motor speed, verbal fluency, attention, and executive function [14]. Thus, BACS and the additionally chosen tests assess cognitive domains, which are known or in question to be altered in patients with diabetes [5]. Moreover, BACS provides age-specific norms derived from the same norm sample for all its subtests, and comparable norms were also available for the additional cognition tests [15, 16].

### 2.3. Brief Assessment of Cognition in Schizophrenia (BACS) Test

#### 2.3.1. Verbal Memory (List Learning)

The interviewer read a list with 15 different words to the participant, who was asked to repeat from memory as many words as possible. This procedure was repeated five times. Measures are the total number of words recalled correctly (range 0 to 75).

#### 2.3.2. Working Memory (Digit Sequencing Task)

The interviewer read a disordered row of numbers with increasing length to the participant, who was asked to repeat the numbers in the correct order from the lowest to the highest number. Measures are the number of correct responses (range 0 to 28) and the longest sequence recalled correctly (range 0 to 8).

#### 2.3.3. Motor Speed (Token Motor Task)

Individuals were asked to put 100 plastic tokens, two at a time, into one bowl within 60 seconds. Measures are the number of tokens correctly placed into the bowl within 60 seconds (range 0 to 100).

#### 2.3.4. Verbal Fluency

*(1) Semantic Fluency*. Individuals were given 60 seconds to name as many words as possible of a defined category, namely, animals. Measures are the total number of correctly named words in the category within 60 seconds.

*(2) Letter Fluency*. Individuals were asked to name as many words as possible with a given initial letter within 60 seconds. Initial letters were F and S. Measures are the number of words named correctly.

#### 2.3.5. Attention and Speed of Information Processing (Symbol Coding)

During this paper-based test, participants were asked to match numerals from 1 to 9 to symbols according to a given code within 90 seconds. Measures are the number of correct assigned numerals (range 0 to 110).

#### 2.3.6. Executive Function (Tower of London)

Individuals were presented two pictures simultaneously, which showed three colored balls (blue, red, and green) differently arranged on three pegs. Individuals were then asked to give the lowest number of moves to make the arrangement of balls identical on both pictures. The test includes 20 trails with increasing difficulty. If the participant consecutively gave five wrong answers, the test was discontinued. If all 20 items were answered correctly, two additional tasks of greater difficulty were presented. Measures are the number of correct answers (range 0 to 22).

Each subtest and BACS composite score was analyzed separately.

### 2.4. Additional Cognition Tests

#### 2.4.1. Executive Function (Trail Making Test (TMT) A/B)

Individuals were asked to connect as quickly as possible numerals (1 to 25) in correct ascending sequence or numerals and letters (1-A to 13) in the correctly increasing numeric and alphabetical row. There was no time limit. Measures are the time used by the participant [17]. Values were given as *T* values.

#### 2.4.2. Social Cognition (Pictures of Facial Affect)

28 pictures of faces with different emotional states were shown to the individuals. Individuals were asked to assign every facial expression to one of the seven given emotions (happiness, sadness, fear, anger, disgust, surprise, and uninvolved). There was no time limit. Measures are the number of correct assignments (range 0 to 28) [16]. Values were given as *T* values.

#### 2.4.3. Crystallized Intelligence (Multiple Choice Word Test B (MWT-B))

During this paper-pencil test, individuals were presented five similar sounding words in a row. Only one of these words was an existing word, which had to be underlined. There was no time limit. Measures are the number of correctly identified words (range 0 to 37) [18]. Values were given as IQ values.

Test scores of all subtests were transformed into *z*-scores according to age-specific norms [14].

### 2.5. Endocrine and Metabolic Tests

#### 2.5.1. Glucagon Stimulation Test

Glucagon-stimulated C-peptide secretion capacity was assessed using the glucagon stimulation test. Blood samples were taken before (0 min) and after (6 min) an injection of 1 mg glucagon (GlucaGen, Novo Nordisk, Mainz, Germany) within 60 s into the antecubital vein. The difference between the C-peptide concentration at 6 min and 0 min was used to determine the glucagon-stimulated C-peptide secretion capacity [13].

#### 2.5.2. Hyperinsulinemic-Euglycemic Clamp Test

Whole-body insulin sensitivity (*M* value) was assessed from the hyperinsulinemic-euglycemic clamp test [13]. Briefly, participants were injected a priming insulin dose (10 mU × body weight (kg)^−1^ × min^−1^ for 10 min) followed by a continuing insulin infusion (1.5 mU × body weight (kg)^−1^ × min^−1^) of short-acting human insulin (Insuman® Rapid, Sanofi-Aventis, Frankfurt am Main, Germany). Every 5 minutes, blood glucose levels were measured and maintained at 5 mmol/l with an intravenous infusion of 20% of glucose. *M* value was assessed as space-corrected mean glucose infusion rate during the last 30 minutes of the clamp.

#### 2.5.3. Oral Glucose Tolerance Test (OGTT)

In healthy individuals, the OGTT was conducted according to the current guidelines of the American Diabetes Association [19]. In the morning, after an overnight fast (≥8 hours), participants drank within 5 minutes 75 g of glucose dissolved in 300 ml water. Blood samples were taken before 0 min and 30 min, 60 min, 90 min, and 120 min after drinking the glucose solution. During the test, participants were asked not to eat, drink, smoke, or perform exercise.

### 2.6. Laboratory Analyses

Fasting blood glucose was measured by EKF Biosen C-Line glucose analyzer (EKF diagnostic GmbH, Barleben, Germany), C-peptide was analyzed on a chemoluminimetric microparticle enzyme-immunoassay (Immulite2000 XPi, Siemens, Erlangen, Germany) or by radioimmunoassay (Millipore, St. Charles, MO, USA), HbA1c was measured on a Variant II (Bio-Rad, Munich, Germany), and high-sensitivity C-reactive protein (hsCRP) was measured on a Roche/Hitachi c 311 analyzer (Roche, Basel, Switzerland) [13].

### 2.7. Statistical Analyses

For descriptive statistics, mean and standard deviation for continuous and percentages for categorical variables were computed, respectively. For comparing the cognition test outcome measures between individuals with type 1 and type 2 diabetes and metabolically healthy individuals, linear regression models with diabetes type (including type 1 and type 2 diabetes as well as healthy individuals), age, sex, and crystallized intelligence as independent variables were used. The models were chosen to adjust differences in cognition test outcome measures between groups for relevant confounders because of the GDS as an observational study. To avoid collinearities, treatment was not included as covariate because all individuals with type 1 diabetes received insulin, whereas the majority of individuals with type 2 diabetes were treated with oral glucose-lowering medications. Because of this strong correlation between diabetes type and treatment, we only included diabetes type, which thereby also serves as a surrogate of treatment. To additionally account for potential confounders, linear regression analyses were performed separately for subgroups of individuals with type 1 and type 2 diabetes with cognition test outcome measures as dependent variable and demographic (age, sex), anthropometric (BMI), metabolic (*M* value, HbA1c), inflammation (hsCRP), and intelligence (MWT-B) parameters as independent variables. Of note, linear regression analyses adjusted for additional confounders were only performed in individuals with recently diagnosed diabetes due to the limitation of the number of cases in the other groups. To visualize the results of the associations of cognition test results and potential risk factors, we prepared additional figures showing the effect of anthropometric (BMI) and crystallized intelligence (MWT-B) on verbal memory test results. Thus, we predicted verbal memory for arbitrary individuals with varying BMI or MWT-B, but with fixed values for the other included covariates (*M* value, HbA1c, hsCRP, age, and sex). *P* values of two-sided tests (*P* < 0.05) were accepted to indicate significant differences. SAS, version 9.3 (SAS, Institute, Cary, NC, USA), was used for all analyses.

## 3. Results

In all groups, male participants predominated (Table 1). Healthy individuals and patients with recently diagnosed type 2 diabetes were about 50 years old, whereas patients with recently diagnosed type 1 diabetes were 35 years old. Healthy individuals and type 2 diabetes patients were overweight to obese, whereas type 1 diabetes patients were only slightly overweight, recently as well as five years after diagnosis. Overall, all individuals with diabetes had very good metabolic control based on HbA1c and fasting blood glucose concentrations. Insulin secretion was lower in patients with type 1 diabetes than in patients with type 2 diabetes or healthy individuals. In contrast, insulin sensitivity was lower in patients with type 2 diabetes than in patients with type 1 diabetes or healthy individuals.

Cognitive testing comprising different cognitive functions revealed no general differences between diabetes patients and healthy individuals (Table 2).

However, individuals with recently diagnosed type 2 diabetes showed a lower score in verbal memory compared to healthy individuals, when adjusting for age, sex, and crystallized intelligence which indicates one's lifetime intellectual achievement (Table 3). Among participants with a known diabetes duration of five years, individuals with type 2 diabetes also exhibited impaired verbal memory compared to individuals with type 1 diabetes. There was no evidence for differences in cognition tests on working memory, motor speed, verbal fluency, attention and speed of information processing, executive function, and social cognition between individuals with recently diagnosed type 1 and type 2 diabetes and metabolically healthy individuals as well as between type 1 and type 2 participants with a known diabetes duration of five years.

**Figure 1:**
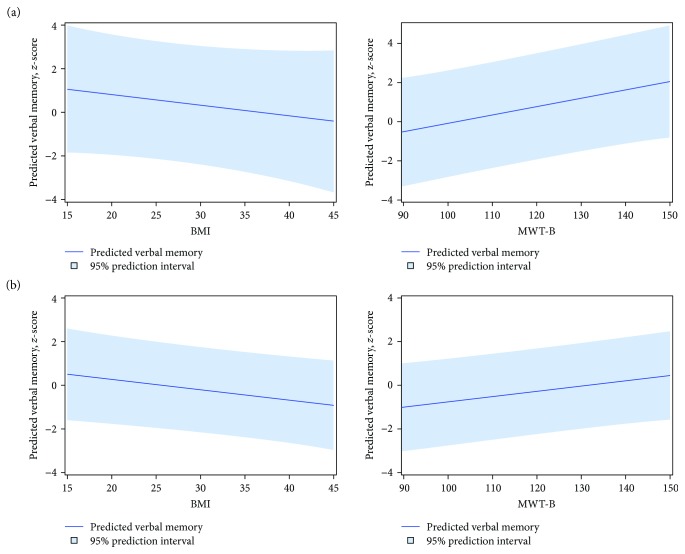
Associations of verbal memory test results with body mass index (BMI) and crystallized intelligence as assessed by multiple choice word test B (MWT-B) in individuals with recently diagnosed type 1 (a) and type 2 diabetes (b). Verbal memory was predicted for arbitrary individuals with varying BMI or MWT-B, but with fixed values for the other included covariates (*M* value, HbA1c, hsCRP, age, and sex).

Among individuals with recently diagnosed type 2 diabetes, a higher score for the MWT-B-test, a measure of crystallized intelligence, was associated with better performance on verbal memory (Table 4, Figure 1(b)). Furthermore, higher BMI was related to a lower number of remembered words reflecting a lower performance in the verbal memory test (Table 4, Figure 1(b)). There was no evidence for an association between age, sex, HbA1c, hsCRP, and insulin sensitivity with verbal memory among individuals with recently diagnosed type 2 diabetes (Table 4). A higher MWT-B test score was also associated with a better outcome of verbal memory in individuals with recently diagnosed type 1 diabetes (Figure 1(a)). Additionally, male sex was associated with poorer performance in verbal memory. Age, BMI, HbA1c, hsCRP, and insulin sensitivity were not associated with verbal memory in individuals with recently diagnosed type 1 diabetes (Table 4). However, increasing BMI was associated with decreasing verbal memory also in patients with recently diagnosed type 1 diabetes (Figure 1(a)).

## 4. Discussion

This study indicates that differences in neurocognitive function, specifically in verbal memory, are present shortly after diagnosis of type 2 diabetes. Five years after diabetes diagnosis, differences in verbal memory were observed between type 2 and type 1 diabetes. A higher BMI among individuals with recently diagnosed type 2 diabetes was associated with a worse performance in verbal memory. While in recently diagnosed type 1 diabetes, the male sex was associated with an impaired verbal memory.

Most of the studies on cognitive function in diabetes focused on participants with longer diabetes duration especially in type 2 diabetes [2, 4, 21, 22]. In one study, cognitive testing was performed 3-4 years after diabetes diagnosis. In line with our study, the authors also found a lower performance in verbal memory in particular in immediate and incidental memory in patients with type 2 diabetes compared to healthy individuals. However, individuals with type 1 diabetes were not included [1]. In two additional studies, cognition testing was performed in the early state of diabetes, but in both studies, the exact diabetes duration is not given [7, 23]. While one of these studies did not specifically test for verbal memory [7], the other did not find a difference in memory between individuals with incident type 2 diabetes and healthy individuals [23]. Also in these two studies, individuals with type 1 diabetes were not included.

There are inconsistent findings on the alterations in subdomains of memory function in individuals with type 2 diabetes. While recent findings suggest a reduced performance in delayed memory [23, 24], other studies observed a decline in immediate memory [1], which is in line with our results.

The lack of differences in other cognitive functions between recently diagnosed type 1 and type 2 diabetes and healthy individuals might be due to the overall good glycemic control, the short disease duration, and the young age of the enrolled participants. An interaction between severity of cognitive problems and age has been described among individuals with type 2 diabetes, with cognitive deficits being more pronounced in individuals older than 60–65 years [25]. Moreover, time of disease duration has been associated with the outcome and intensity of cognitive impairment in individuals with diabetes [1, 24, 26]. This is in line with our findings, showing impaired verbal memory in patients with type 2 diabetes compared to those with type 1 diabetes after five years.

Obesity is not only a strong risk factor for type 2 diabetes, but may also associate with lower cognitive function in metabolically healthy individuals, including the domain of memory function as shown by a previous study [10]. In the present study, we observed an inverse association between BMI and outcome of verbal memory among individuals with recently diagnosed type 2 diabetes. Previous observations in type 2 diabetes indicated that obesity itself contributes to functional and structural brain changes [22]. For example, reduction of hippocampal volume was observed with increasing BMI [9]. The mechanisms by which obesity could cause cognitive decline seem to be multifactorial and are not yet completely understood. A possible mechanism refers to obesity-associated inflammation and suggests that increased expression of proinflammatory cytokines, for example, interleukin-6 and tumor necrosis factor-*α*, is associated with cognitive decline [27]. Furthermore, higher BMI was associated with reduced cerebral blood flow velocity in individuals with type 2 diabetes [28]. Additionally, obesity-related peripheral insulin resistance has been also associated with lower transport of insulin across the blood brain barrier, which would lead to cognitive impairment [29]. Moreover, studies in obese Zucker rats demonstrated reduction in plasma membrane glucose transporter (GLUT)-4 in the hippocampus leading to decreased local glucose uptake in this brain area, which plays a crucial role for learning and memory [30]. Also in humans, peripheral insulin resistance was associated with a reduced regional cerebral glucose metabolism especially in the left medial temporal lobe predicting worse immediate and delayed memory performance. Loss of neuronal function leading to mitochondrial damage as well as amyloid deposition in the brain is a mechanism possibly explaining the association between insulin resistance and impaired cognitive function [31]. Hence, obesity may alter brain morphology and function, which may translate into impaired cognitive performance among individuals with diabetes [9].

Unlike previous studies, we observed an association between male sex and worse performance in verbal memory among individuals with recently diagnosed type 1 diabetes. Previous studies only related male sex to a poorer performance in measures of learning [21] as well as in verbal fluency, mental tasks, and processing speed in individuals with type 2 diabetes [4]. The studies observing differences between sex mainly concluded that not sex itself but rather factors being associated with sex, for example, poorer glycemic control being observed in men than in women, might explain the differences in cognitive outcome [32]. Furthermore, a few additional studies hypothesized a neuronal protective effect of estrogen [33], which might explain the differences in cognitive performance between men and women. However, most studies did not report differences for sex, but used this variable as a confounder.

The influence of metabolic control on cognitive decline has been controversially discussed [1, 7, 8, 26]. Our results do not support an association between HbA1c or insulin sensitivity and verbal memory in individuals with recent-onset diabetes. This is in line with studies concluding that a strict metabolic control and short diabetes duration might be possible causes of missing effects [1], whereas a diabetes duration of more than 5 years and an increasing HbA1c were found to be associated with a lower cognitive performance [7, 24, 26]. The overall good metabolic control of all patients in the present study likely reflects intensive diabetes treatment by either insulin or oral glucose-lowering medication. Pharmacological treatment may increase the risk of hypoglycemia, which in turn could accelerate cognitive decline [34]. Of note, the postulated relationship between higher frequency of hypoglycemia and cognitive impairment has not been demonstrated in all studies [11, 12]. On the other hand, medication leading to improved insulin sensitivity could have beneficial effects on cognitive function [34], which reflects the association between insulin resistance and impaired cognitive function, observed in some studies [29, 31]. In contrast to these studies, the present study did not show an association between insulin resistance and cognitive function. This discrepancy may result from divergent measurements of insulin resistance. In previous studies, insulin resistance was mainly assessed by surrogate parameters [7], whereas the present study employed the gold standard for assessment of insulin sensitivity.

Strengths of our study are the intensive metabolic phenotyping of the study population and the inclusion of both individuals with recently diagnosed type 1 and type 2 diabetes as well as metabolically healthy individuals. In addition, all participants underwent a comprehensive neurocognitive test battery, measuring their cognitive performance across multiple domains. The present study is limited by the cross-sectional analyses. Furthermore, individuals with recently diagnosed type 1 and type 2 diabetes and those with a known diabetes duration of five years represent different cohorts. However, both groups followed the identical protocols of metabolic and cognition tests, thereby allowing for comparison of the results. Moreover, due to the rather small number of participants with a known diabetes duration of five years, associations between potential risk factors and parameters of cognitive function were not tested in this group. Furthermore, at the time of analyses, there was no healthy control group available for patients with a known diabetes duration of five years. By nature of the diseases, the age range differed between the two groups of recent onset diabetes patients. The age of the control group is appropriate for comparison with the type 2 diabetes group, but not with the type 1 diabetes group. In order to minimize the influence of age, a potential risk factor for cognitive impairment, the cognition test outcomes were adjusted for age. Finally, all participants showed overall very good glycemic control, which limits the generalizability of our results. However, it is conceivable that worsening of glycemic control might rather aggravate cognitive impairment.

In conclusion, recently diagnosed type 2 diabetes is associated with cognitive impairment in verbal memory upon adjustment for demographic confounders with higher BMI as a possible risk factor. The results of our study, therefore, point to the impairment of cognitive function as an early diabetes-related complication that deserves particular attention in the treatment of patients with diabetes.

## Figures and Tables

**Table 1:** Characteristics of the study participants.

	Metabolically healthy individuals, *n* = 42	Individuals with recently diagnosed diabetes, *n* = 201		Individuals with a known diabetes duration of five years, *n* = 110	
	Healthy individuals	Type 1 diabetes	Type 2 diabetes	Type 1 diabetes	Type 2 diabetes
Number (*n*)	42	82	119	45	65
Male (*n*/%)	35 (83)	47 (57)	76 (64)	27 (60)	41 (63)
Age (years)	49 ± 12	35 ± 10	52 ± 9	41 ± 13	59 ± 10
Body mass index (kg/m^2^)	29.0 ± 6.1	25.3 ± 3.8	32.0 ± 5.7	25.1 ± 3.0	32.1 ± 5.5
Time since diagnosis (months)	—	5.9 ± 3.0	5.8 ± 3.4	71.4 ± 13.7	70.8 ± 12.8
HbA1c (%)	5.3 ± 0.3	6.3 ± 1.1	6.4 ± 0.9	6.9 ± 1.0	6.9 ± 1.1
HbA1c (mmol/mol)	34 ± 3	45 ± 11	46 ± 10	52 ± 11	52 ± 12
Fasting glucose (mg/dl)	91 ± 8	123 ± 38	131 ± 26	142 ± 49	157 ± 46
Fasting C-peptide (ng/ml)	1.9 (1.4; 2.5)	0.9 (0.5; 1.4)	3.1 (2.4; 4.0)	0.2 (0.1; 0.6)	2.8 (2.3; 4.1)
C-peptide secretion capacity (ng/ml)	3.8 (2.7; 4.9)	0.6 (0.2; 1.4)	3.0 (2.1; 4.0)	0.1 (0.0; 0.4)	3.2 (2.1; 4.4)
*M* value (mg × kg^−1^ ^×^ min^−1^)	10.3 (8.5; 12.4)	8.3 (6.8; 10.4)	5.4 (4.2; 7.4)	7.5 (6.1; 8.3)	5.3 (3.8; 6.6)

Data are *n* (%), mean ± SD, or median (25th and 75th percentiles). According to the STROBE guidelines, no *P* value is given [20]. STROBE: Strengthening the Reporting of Observational Studies in Epidemiology.

**Table 2:** Unadjusted cognition test results in participants with recently diagnosed type 1 and type 2 diabetes and metabolically healthy individuals as well as in participants with a known type 1 and type 2 diabetes duration of five years.

	Metabolically healthy individuals		Individuals with recently diagnosed diabetes				Individuals with a known diabetes duration of five years			
	Healthy individuals		Type 1 diabetes		Type 2 diabetes		Type 1 diabetes		Type 2 diabetes	
	*n* ^$^	Mean ± SD	*n* ^$^	Mean ± SD	*n* ^$^	Mean ± SD	*n* ^$^	Mean ± SD	*n* ^$^	Mean ± SD
Verbal memory^†^	42	0.3 ± 1.2	82	0.2 ± 1.5	119	−0.3 ± 1.0	45	0.3 ± 1.0	63	−0.5 ± 1.2
Digit sequencing^†^	42	0.1 ± 1.1	81	−0.1 ± 1.0	118	−0.1 ± 1.1	45	−0.1 ± 1.1	64	0.1 ± 1.1
Token motor task^†^	42	0.3 ± 1.1	82	0.3 ± 0.9	119	0.3 ± 1.0	45	0.5 ± 1.0	63	0.1 ± 1.1
Verbal fluency^†^	42	−0.1 ± 1.1	82	−0.1 ± 1.2	118	−0.04 ± 1.1	45	−0.04 ± 0.9	64	−0.3 ± 1.0
Symbol coding score^†^	42	−0.2 ± 0.8	82	−0.002 ± 1.0	119	−0.3 ± 1.0	45	−0.1 ± 0.9	64	−0.5 ± 1.2
Tower of London^†^	42	0.1 ± 0.8	82	0.3 ± 0.9	119	0.1 ± 0.8	45	0.2 ± 0.7	64	−0.1 ± 1.0
BACS composite score^†^	42	0.1 ± 1.1	81	0.2 ± 1.1	118	−0.1 ± 1.0	45	0.2 ± 0.9	62	−0.3 ± 1.1
TMT_A^‡^	42	49.3 ± 9.2	82	51.4 ± 10.7	119	48.9 ± 11.2	45	51.0 ± 9.9	64	48.5 ± 8.4
TMT_B^‡^	42	51.2 ± 10.4	82	52.3 ± 9.4	118	50.9 ± 9.9	45	50.1 ± 9.0	62	48.8 ± 8.5
Pictures of facial affect^‡^	42	42.4 ± 10.7	82	47.3 ± 10.9	118	44.0 ± 11.4	45	44.7 ± 9.9	65	42.9 ± 9.6
MWT-B^∗^	41	115.1 ± 12.1	82	115.0 ± 14.0	119	118.9 ± 14.7	44	115.6 ± 12.6	64	114.4 ± 12.8

Data are *n*. ^†^Mean ± SD of *z*-score values or ^‡^mean ± SD of *T*-score values. ^∗^Mean ± SD of IQ values. According to the STROBE guidelines, no *P* value is given [20]. ^$^Given that few participants failed to finish all cognitive tests, the number of participants who performed completely cognitive testing is given for each test. BACS: Brief Assessment of Cognition in Schizophrenia; MWT-B: multiple choice word test B; TMT_A/B: trail making test A/B; STROBE: Strengthening the Reporting of Observational Studies in Epidemiology.

**Table 3:** Comparison of cognition tests between recently diagnosed type 1 and type 2 diabetes and healthy individuals and between participants with a known type 1 and type 2 diabetes duration of five years after adjusting for crystallized intelligence, age, and sex.

	Individuals with recently diagnosed diabetes and metabolically healthy individuals						Individuals with a known diabetes duration of five years	
	Type 2 diabetes versus type 1 diabetes		Type 2 diabetes versus healthy individuals		Type 1 diabetes versus healthy individuals		Type 2 diabetes versus type 1 diabetes	
	*β* (95% CI)	*P* value	*β* (95% CI)	*P* value	*β* (95% CI)	*P* value	*β* (95% CI)	*P* value
Verbal memory	−0.12 (−0.56; 0.33)	0.602	−0.49 (−0.93; −0.05)	*0.029*	−0.37 (−0.89; 0.14)	0.156	−0.81 (−1.44; −0.18)	*0.012*
Digit sequencing	0.02 (−0.37; 0.41)	0.917	−0.20 (−0.58; 0.18)	0.306	−0.22 (−0.67; 0.23)	0.339	0.34 (−0.22; 0.90)	0.227
Token motor task	−0.23 (−0.58; 0.13)	0.204	−0.06 (−0.42; 0.29)	0.716	0.16 (−0.25; 0.58)	0.435	−0.21 (−0.79; 0.38)	0.486
Verbal fluency	−0.03 (−0.43; 0.38)	0.897	−0.12 (−0.52; 0.28)	0.561	−0.09 (−0.57; 0.38)	0.702	−0.24 (−0.76; 0.27)	0.347
Symbol coding score	0.07 (−0.26; 0.40)	0.680	−0.12 (−0.45; 0.21)	0.483	−0.19 (−0.57; 0.20)	0.343	−0.18 (−0.71; 0.34)	0.487
Tower of London	−0.08 (−0.39; 0.23)	0.606	0.03 (−0.28; 0.33)	0.864	0.11 (−0.25; 0.47)	0.557	−0.12 (−0.60; 0.36)	0.615
BACS composite score	−0.08 (−0.42; 0.27)	0.659	−0.26 (−0.60; 0.08)	0.130	−0.19 (−0.59; 0.22)	0.363	−0.32 (−0.83; 0.19)	0.221
TMT_A	0.52 (−3.42; 4.47)	0.794	−0.55 (−4.47; 3.36)	0.781	−1.08 (−5.68; 3.53)	0.646	−1.75 (−6.83; 3.33)	0.495
TMT_B	0.81 (−2.63; 4.26)	0.642	−1.43 (−4.85; 1.99)	0.411	−2.24 (−6.26; 1.78)	0.273	0.23 (−4.61; 5.07)	0.925
Pictures of facial affect	1.72 (−2.15; 5.59)	0.382	2.00 (−1.85; 5.85)	0.307	0.28 (−4.25; 4.81)	0.904	0.17 (−5.20; 5.53)	0.096

Data are mean differences (*β*), 95% confidence intervals (CI), and *P* values from linear regression with MWT-B, as a marker of crystallized intelligence, age, and sex as independent variables and cognition tests as a dependent variable. Italicized print indicates *P* < 0.05. BACS: Brief Assessment of Cognition in Schizophrenia; TMT_A/B: trail making test A/B.

**Table 4:** Associations of verbal memory test results with *M* value, hsCRP, HbA1c, crystallized intelligence, BMI, age, and sex in individuals with recently diagnosed type 1 and type 2 diabetes.

	Type 1 diabetes		Type 2 diabetes	
Independent variables	*β* (95% CI)	*P* value	*β* (95% CI)	*P* value
*M* value	0.04 (−0.07; 0.16)	0.475	0.03 (−0.06; 0.12)	0.554
hsCRP	2.05 (−0.31; 4.41)	0.087	0.43 (−0.22; 1.08)	0.193
HbA1c	−0.17 (−0.51; 0.16)	0.308	0.01 (−0.22; 0.24)	0.938
Crystallized intelligence	0.04 (0.02; 0.07)	*0.003*	0.02 (0.01; 0.04)	*0.001*
BMI	−0.05 (−0.14; 0.05)	0.310	−0.05 (−0.09; −0.01)	*0.025*
Age	−0.02 (−0.06; 0.02)	0.256	−0.01 (−0.04; 0.01)	0.362
Sex (male)	1.22 (0.51; 1.93)	*0.001*	0.003 (−0.46; 0.46)	0.989

Data are regression coefficients (*β*), 95% confidence intervals (CI), and *P* values from linear regression with *M* value, hsCRP, HbA1c, crystallized intelligence, BMI, age, and sex as independent variables and verbal memory as a dependent variable. Italicized print indicates *P* < 0.05. hsCRP: high-sensitivity C-reactive protein; BMI: body mass index.

## Data Availability

A request and transfer process has been established so that researches may apply for data by contacting the study coordinators via email (GDS@ddz.uni-duesseldorf.de). Once approved by the steering committee, the requesting researcher and the principal investigator of GDS sign a contract on the terms and conditions of data transfer and transmission of results back to the German Diabetes Center.
